# Intra-amniotic inflammation and birth weight in pregnancies with preterm labor with intact membranes: A retrospective cohort study

**DOI:** 10.3389/fped.2022.916780

**Published:** 2022-11-28

**Authors:** Jana Matulova, Marian Kacerovsky, Radka Bolehovska, Rudolf Kukla, Pavel Bostik, Klara Kolarova, Simona Frydrychová, Bo Jacobsson, Ivana Musilova

**Affiliations:** ^1^ Department of Non-Medical Studies, Faculty of Medicine in Hradec Kralove, Charles University, Hradec Kralove, Czech Republic; ^2^ Department of Obstetrics and Gynecology, University Hospital Hradec Kralove, Faculty of Medicine in Hradec Kralove, Charles University, Hradec Kralove, Czech Republic; ^3^ Biomedical Research Center, University Hospital Hradec Kralove, Hradec Kralove, Czech Republic; ^4^ Institute of Clinical Microbiology, University Hospital Hradec Kralove, Faculty of Medicine in Hradec Kralove, Charles University, Hradec Kralove, Czech Republic; ^5^ Department of Obstetrics and Gynecology, Institute of Clinical Science, Sahlgrenska Academy, University of Gothenburg, Gothenburg, Sweden; ^6^ Department of Obstetrics and Gynecology, Region Västra Götaland, Sahlgrenska University Hospital, Gothenburg, Sweden; ^7^ Department of Genetics and Bioinformatics, Domain of Health Data and Digitalization, Institute of Public Health, Oslo, Norway

**Keywords:** amniocentesis, amniotic fluid, estimated fetal weight, fetal growth, intergrowth, intra-amniotic inflammation, microbial invasion of the amniotic cavity, preterm birth

## Abstract

**Objective:**

To assess the association between newborn birth weight and the presence of intra-amniotic infection, presence of sterile intra-amniotic inflammation, and absence of intra-amniotic inflammation in pregnancies with preterm labor with intact membranes.

**Methods:**

A total of 69 pregnancies with preterm labor with intact membranes between gestational ages 22 + 0 and 34 + 6 weeks who delivered within seven days of admission were included in this retrospective cohort study. Transabdominal amniocentesis to determine the presence of microorganisms and/or their nucleic acids in amniotic fluid (through culturing and molecular biology methods) and intra-amniotic inflammation (according to amniotic fluid interleukin-6 concentrations) were performed as part of standard clinical management. The participants were further divided into three subgroups: intra-amniotic infection (presence of microorganisms and/or nucleic acids along with intra-amniotic inflammation), sterile intra-amniotic inflammation (intra-amniotic inflammation alone), and without intra-amniotic inflammation. Birth weights of newborns were expressed as percentiles derived from the INTERGROWTH-21st standards for (i) estimated fetal weight and (ii) newborn birth weight.

**Results:**

No difference in birth weights, expressed as percentiles derived from the standard for estimated fetal weight, was found among the women with intra-amniotic infection, with sterile intra-amniotic inflammation, and without intra-amniotic inflammation (with infection, median 29; with sterile inflammation, median 54; without inflammation, median 53; *p = *0.06). Differences among the subgroups were identified in the birth weight rates, expressed as percentiles derived from the standard for estimated fetal weight, which were less than the 10th percentile (with infection: 20%, with inflammation: 13%, without inflammation: 0%; *p* = 0.04) and 25th percentile (with infection: 47%, with inflammation: 31%, without inflammation: 9%; *p* = 0.01). No differences among the subgroups were observed when percentiles of birth weight were derived from the birth weight standard.

**Conclusions:**

The presence of intra-amniotic inflammatory complications in pregnancies with preterm labor with intact membranes prior to the gestational age of 35 weeks was associated with a higher rate of newborns with birth weight less than the 10th and 25th percentile, when percentiles of birth weight were derived from the standard for estimated fetal weight.

## Introduction

Preterm labor with intact membranes (PTL), a clinical phenotype of spontaneous preterm delivery, accounts for approximately one-third of all preterm deliveries (1–3). This serious pregnancy complication, considered as one of “the great obstetrical syndromes” (4, 5), still represents an unsolved problem of current perinatology with global medical and socioeconomic impacts (6).

Almost half of PTL cases are complicated by intra-amniotic inflammation (7, 8), a condition characterized by the elevation of a broad spectrum of pro- and anti-inflammatory mediators in the amniotic fluid (9). This pregnancy complication has two phenotypes depending on the presence or absence of microorganisms and/or their nucleic acids in amniotic fluid: intra-amniotic infection and sterile intra-amniotic inflammation (7, 10, 11). Irrespective of its nature, intra-amniotic inflammation in PTL is associated with (i) a lower gestational age at delivery (7, 8, 12); (ii) a higher rate of Apgar score <7 at 5 and 10 min (12); (iii) a higher prevalence of *Ureaplasma* spp. in the cervix (8), (iv) a higher frequency of acute inflammatory lesions in the placenta (7); and (v) a higher rate of composite neonatal morbidity (7).

Since the presence of intra-amniotic infection and sterile intra-amniotic inflammation in PTL is associated with a lower gestational age at delivery (7, 8, 12), it is evident that the newborn birth weight from these pregnancies is lower than that of those without intra-amniotic inflammation. However, the possible effect intra-amniotic inflammation on the fetal growth cannot be fully excluded owing to the following previously published data: (i) intra-peritoneal administration of lipopolysaccharide in the second half of pregnancy resulted to reduction of birth weight in a rat animal model (13, 14); (ii) intra-amniotic administration of endotoxin (*Escherichia coli*) decreased birth weight in a rat animal model (15); (iii) a higher expression of the gene for activator protein-1, a transcription factor in the third trimester, predicted a lower birth weight (16); (iv) elevated maternal systemic concentration of interleukin 17A during pregnancy was associated with low birth weight (17); (v) seropositivity for *Helicobacter pylori* in the third trimester was related to a higher rate of fetal growth restriction (18); and (vi) periodontal disease incidence/progression during pregnancy was associated with higher frequency of newborns with smaller birth weight for gestational age (19).

Our group recently reported that intra-amniotic infection and sterile intra-amniotic inflammation were associated with lower birth/aborted weight of newborns in pregnancies with cervical insufficiency with prolapsed fetal membranes (20) but not in pregnancies complicated by preterm prelabor rupture of membranes (21). However, there is a lack of information on whether intra-amniotic infection and sterile intra-amniotic inflammation affect fetal growth in pregnancies complicated by PTL.

Therefore, a retrospective study on women with singleton pregnancies complicated by PTL, delivering within seven days from admission (to eliminate the possible effects of corticosteroids and other conditions that might affect fetal growth during a long latency between amniocentesis and delivery) was conducted with the following goals: (i) to assess the difference in birth weight, expressed as percentiles derived from standards for estimated fetal weight and newborn birth weight, between the subgroups of those with intra-amniotic infection, with sterile intra-amniotic inflammation, and without intra-amniotic inflammation; and (ii) to compare the rates of birth weights that are less than the 1st, 3rd, 10th, and 25th percentiles among the subgroups with intra-amniotic infection, with sterile intra-amniotic inflammation, and without intra-amniotic inflammation.

## Methods

This retrospective study included pregnant women admitted to the Department of Obstetrics and Gynecology of the University Hospital Hradec Kralove, Czech Republic, between March 2017 and November 2021, who met the following criteria: (i) age ≥18 years; (ii) singleton pregnancy; (iii) gestational age between 22 + 0 and 34 + 6 weeks; (iv) PTL with delivery within seven days of admission; and (v) underwent amniocentesis to assess microbial invasion of the amniotic cavity and intra-amniotic inflammation. The exclusion criteria were as follows: (i) pregnancy-related and other medical complications (chronic hypertension, gestational hypertension, preeclampsia, pregestational diabetes mellitus, and gestational diabetes mellitus); (ii) congenital or chromosomal fetal abnormalities; (iii) signs of fetal hypoxia at the time of admission; (iv) significant vaginal bleeding; and (v) preterm prelabor rupture of membranes.

Gestational age was determined based on the first-trimester fetal biometry. PTL was defined as the presence of regular uterine contractions (at least two every 10 min) along with a cervical length <15 mm on transvaginal ultrasound or cervical length of 15–30 mm with a positive PartoSure test (Parsagen Diagnostics Inc., Boston, MA, United States) (22). Transabdominal amniocentesis was performed at the time of admission before administering corticosteroids, tocolytics, or antibiotics.

Women with PTL received an antenatal course of corticosteroids (betamethasone) and tocolytic therapy with either intravenous atosiban (for gestational age ≤28 weeks) or oral administration of nifedipine for 48 h. Women diagnosed with intra-amniotic inflammation received clarithromycin intravenously for seven days, unless delivery occurred earlier. Antibiotic treatment was eventually modified based on the results of microbial assessments of amniotic fluid. Women with PTL who were positive for the vaginal-rectal presence of *Streptococcus agalactiae* or did not have these results available received intravenous benzylpenicillin (clindamycin, in case of penicillin allergy) during active labor.

The collection of clinical samples and information from women with PTL was approved by the Institutional Review Board Committee (June 2015; No. 201408 I96L). Written informed consent was obtained from all women. All the women in the study were self-reported as Caucasians. Biological samples (amniotic fluid and cervical fluid) from the women included in this study were used in our previous studies (8, 12, 23).

### Amniotic fluid sampling

Ultrasonography-guided transabdominal amniocentesis was performed before administration of corticosteroids, antibiotics, or tocolytics. Details of amniotic fluid sampling have been previously described (12, 23).

### Assessment of interleukin-6

The concentration of interleukin (IL)-6 in amniotic fluid was assessed using an automated electrochemiluminescence immunoassay method with the immuno-analyzer Cobas e602 (Roche Diagnostics, Basel, Switzerland) (24). The measurable range was 1.5–5,000 pg/ml, which could be extended to 50,000 pg/ml with a 10-fold dilution of the sample. The coefficients of variation for inter- and intra-assay precisions were both <10%.

### *Detection of* Ureaplasma *species,* Mycoplasma hominis*, and* Chlamydia trachomatis *in the amniotic fluid*

Commercial AmpliSens^@^ *C. trachomatis/Ureaplasma/M. hominis*-FRT kit (Federal State Institution of Science, Central Research Institute of Epidemiology, Moscow, Russia) was used to detect DNA from *Ureaplasma species, M. hominis*, and *C. trachomatis* in the amniotic fluid. The details of this specific PCR run in a single PCR tube have been previously described (12, 23).

### Detection of other microorganisms in the amniotic fluid

Details on the detection of microorganisms and/or their nucleic acids besides *Ureaplasma* spp., *Mycoplasma hominis*, and *Chlamydia trachomatis* in the amniotic fluid using non-cultivation methods and aerobic/anaerobic cultivation have been described previously (12, 23).

### Birth weight percentiles

All newborns were weighed immediately after birth, using a calibrated electronic scale. Birth weights were transformed into percentiles derived from INTERGROWTH-21st standards (25–28) for: i) estimated fetal weight (26, 28) and ii) newborn birth weight (27).

### Clinical definitions

**Microbial invasion of the amniotic cavity** was determined based on a positive PCR analysis for *Ureaplasma* spp.*, M. hominis, or C. trachomatis* or a combination of these microorganisms, positive expression of the 16S rRNA gene, positive aerobic/anaerobic cultivation of the amniotic fluid, or a combination of these parameters. **Intra-amniotic inflammation** was defined as an amniotic fluid IL-6 concentration ≥3,000 pg/ml (29). **Intra-amniotic infection** was defined as the presence of microbial invasion of the amniotic cavity and intra-amniotic inflammation. **Sterile intra-amniotic inflammation** was defined as the presence of intra-amniotic inflammation without microbial invasion of the amniotic cavity.

### Statistical analyses

The demographic and clinical characteristics of the patients were compared using the non-parametric Kruskal-Wallis test for continuous variables and the chi-squared test for categorical variables, and the results are presented as median [interquartile range (IQR)] and number (%), respectively. The normality of the data was tested using the Anderson–Darling test. The percentiles of birth weight were not normally distributed; therefore, the non-parametric Jonckheere-Terpstra test was used for the analyses. The Cochran-Armitage test for trends was used to compare birth weight rates among subgroups. Spearman's partial correlation analysis was performed to adjust the results for a potential confounder (antibiotic administration). Differences were considered statistically significant at *p *< 0.05. All *p*-values were determined using two-tailed tests, and all statistical analyses were performed using GraphPad Prism v8 for Mac OS X (GraphPad Software, San Diego, CA, United States) and Statistical Package for Social Sciences (SPSS), version 28.0.0.0, for Windows (SPSS Inc., Chicago, IL, United States).

## Results

In total, 94 women with singleton pregnancies complicated by PTL who delivered within seven days of admission were eligible for the study. Twenty-five women were excluded for the following reasons: i) gestational diabetes mellitus (*n* = 17), ii) preeclampsia (*n* = 4), pregestational diabetes mellitus (*n* = 2), and gestational hypertension (*n* = 2). The remaining 69 women were included in the analysis.

Microbial invasion of the amniotic cavity and intra-amniotic inflammation were observed in 15 (22%) and 47 (68%) women, respectively. The microbial findings from the amniotic fluid were as follows: *Ureaplasma* spp. + *Capnocytophaga ochracea* + *Fusobacterium nucleatum* (*n* = 1), *Lactobacillus* spp. + *Gardnerella vaginalis* (*n* = 1), *Ureaplasma* spp. (*n* = 5), *Streptococcus* spp. (*n* = 1), *Sneathia sanguinegens* (*n* = 1), *Lactococcus lactis* (*n* = 1), *Haemophilus influenzae* (*n* = 1), *Fusobacterium nucleatum* (*n* = 1), *Burkholderia cepacia* (*n* = 1), and non-identifiable bacteria by sequencing (*n* = 2).

Intra-amniotic infection, sterile intra-amniotic inflammation, and absence of intra-amniotic inflammation were found in 15 (22%), 32 (46%), and 22 (32%) women, respectively. None of the women had microbial invasion of the amniotic cavity without intra-amniotic inflammation.

The demographic and clinical characteristics of the study population with respect to the presence of intra-amniotic infection, presence of sterile intra-amniotic inflammation, and absence of intra-amniotic inflammation are shown in Table 1.

**Table 1:** Maternal and clinical characteristics of women with preterm labor with intact membranes prior to gestational age 35 weeks with respect to the presence of intra-amniotic infection, presence of sterile intra-amniotic inflammation, and absence of intra-amniotic inflammation.

Characteristic	The presence of intra-amniotic infection (*n* = 15)	The presence of sterile intra-amniotic inflammation (*n* = 32)	The absence of intra-amniotic inflammation (*n* = 22)	*p*-value
Maternal age [years, median (IQR)]	28 (26–30)	28 (25–31)	28 (23–31)	0.87
Primiparous [number (%)]	6 (40%)	22 (69%)	13 (59%)	0.17
Previous preterm delivery [number (%)]	1 (7%)	2 (6%)	3 (14%)	0.61
Pre-pregnancy body mass index [kg/m^2^, median (IQR)]	23.4 (21.1–29.2)	23.9 (21.7–27.9)	24.7 (22.9–28.3)	0.60
Smoking [number (%)]	4 (27%)	4 (13%)	2 (9%)	0.30
Gestational age at admission [weeks, median (IQR)]	28 + 2 (24 + 1–33 + 1)	26 + 4 (24 + 2–31 + 1)	31 + 6 (29 + 6–33 + 0)	**0**.**007**
Gestational age at delivery [weeks, median (IQR)]	28 + 4 (24 + 2–33 + 1)	26 + 6 (24 + 3–31 + 4)	32 + 1 (29 + 6–33 + 1)	**0**.**005**
Interval from amniocentesis to delivery [days, median (IQR)]	0 (0–3)	1 (0–3)	2 (1–4)	0.22
Delivery within 7 days from amniocentesis [number (%)]	15 (100%)	32 (100%)	22 (100%)	–
Amniotic fluid IL-6 levels at admission [pg/ml, median (IQR)]	50,000 (33,824–50,000)	8,268 (4,525–34,481)	1,364 (741–2,068)	**<0**.**0001**
CRP levels at admission [mg/L, median (IQR)]	37 (15.3–49.6)	10.7 (6.7–17.8)	5.3 (2.5–12.8)	**0**.**002**
WBC count at admission [×10^9^ L, median (IQR)]	16.7 (11.5–19.1)	15.7 (11.9–16.9)	13.8 (10.6–16.1)	0.19
Administration of corticosteroids [number (%)]	12 (80%)	24 (75%)	20 (91%)	0.34
Administration of antibiotics [number (%)]	13 (87%)	28 (88%)	13 (59%)	**0**.**03**
Vaginal delivery [number (%)]	13 (87%)	29 (91%)	17 (77%)	0.39
Birth weight of the newborn [grams, median (IQR)]	1,200 (660–1,840)	955 (670–1,950)	1,880 (1,323–2,155)	**0**.**007**
Apgar score <7; 5 min [number (%)]	8 (53%)	12 (38%)	2 (9%)	**0**.**01**
Apgar score <7; 10 min [number (%)]	7 (47%)	10 (31%)	0 (0%)	**0**.**003**

CRP, C-reactive protein; IL, interleukin; IQR, interquartile range; WBC, white blood cells.

Continuous variables were compared using a nonparametric Kruskal–Wallis test. Categorical variables were compared using the chi-square test. Continuous variables are presented as median (IQR) and categorical as number (%).

Statistically significant results are marked in bold.

### Birth weight expressed as percentiles derived from estimated fetal weight standards

**Figure 1:**
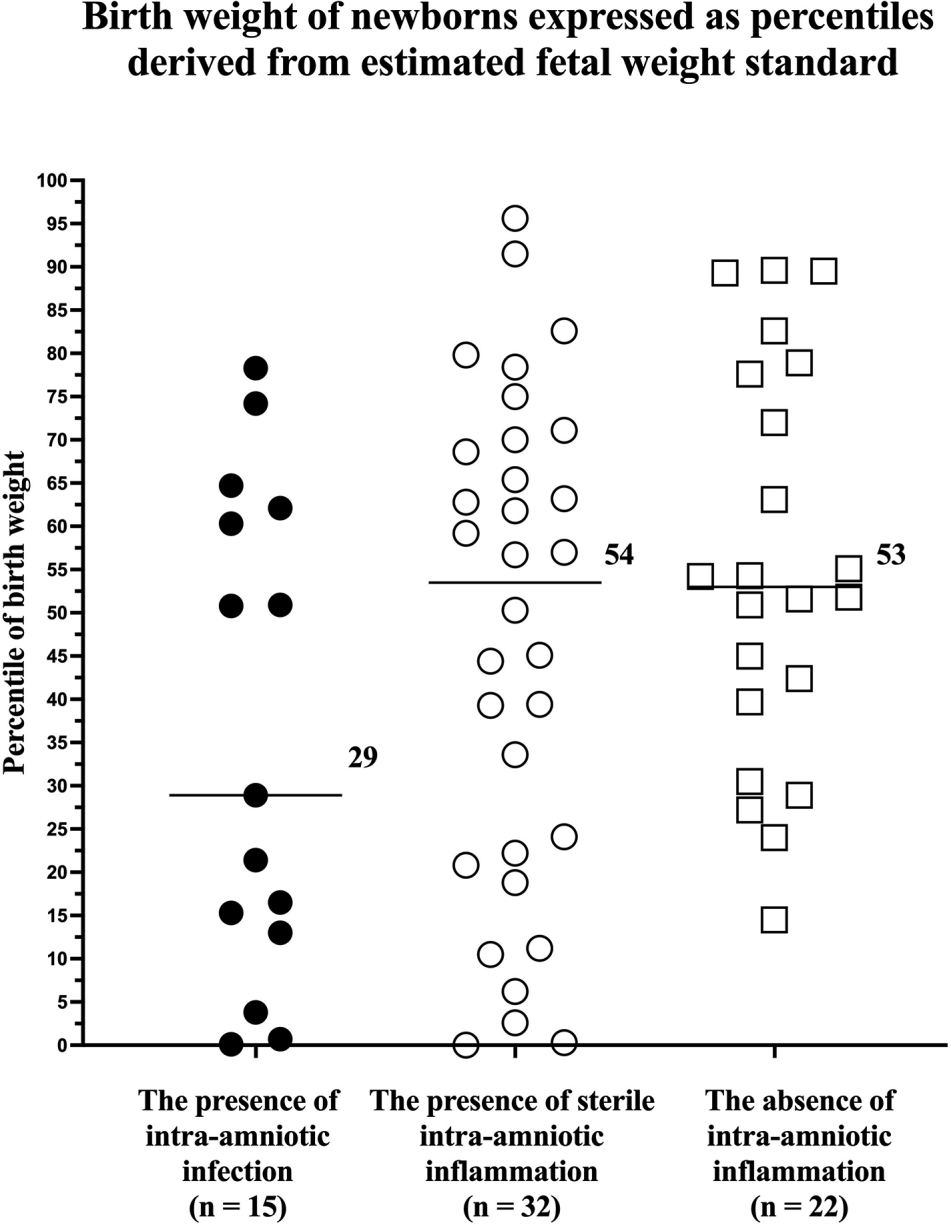
Comparison of newborn birth weight, expressed as percentiles derived from estimated fetal weight standards, from pregnancies with preterm labor with intact membranes prior to a gestational age of 35 weeks with the presence of intra-amniotic infection, presence of sterile intra-amniotic inflammation, and absence of intra-amniotic inflammation. Medians are marked.

No differences were found among the subgroups of women with intra-amniotic infection, with sterile intra-amniotic inflammation, and without intra-amniotic inflammation in percentiles of newborn birth weight (the presence of intra-amniotic infection: median 29, IQR 13–62; the presence of sterile intra-amniotic inflammation: median 54, IQR 21–70; the absence of intra-amniotic inflammation 53, IQR 37–78; *p* = 0.06; Figure 1) and in the rates of the newborn birth weight that were less than the 1st and 3rd percentiles (Table 2). Differences were revealed among the subgroups in the rates of newborn birth weight less than the 10th and 25th percentiles in crude analysis (10th percentile: *p* = 0.04 and 25th percentile: *p* = 0.01, Table 2), as well as in the analysis adjusted for administration of antibiotics (10th percentile: *p = *0.04*;* 25th percentile: *p *= 0.02).

**Table 2:** The rate of newborns with birth weights, expressed as percentiles derived from estimated fetal weight standards, that were less than or equal to the 1st, 3rd, 10th, and 25th percentiles from pregnancies with preterm labor with intact membranes prior to a gestational age of 35 weeks with the presence of intra-amniotic infection, presence of sterile intra-amniotic inflammation, and absence of intra-amniotic inflammation.

	<1st percentile	<3rd percentile	<10th percentile	<25th percentile
The presence of intra-amniotic infection (*n* = 15)	2 (13%)	*p* = 0.09	2 (13%)	*p* = 0.11	3 (20%)	***p* = 0.04** *	7 (47%)	***p* = 0.01** *
The presence of sterile intra-amniotic inflammation (*n* = 32)	2 (6%)		3 (9%)		4 (13%)		10 (31%)	
The absence of intra-amniotic inflammation (*n* = 22)	0 (0%)		0 (0%)		0 (0%)		2 (9%)	

Variables are presented as number (%) and were compared using Cochran-Armitage test for trend.

Statistically significant results are marked in bold.

* The result remains significant after the adjustment for potential confounder (antibiotic treatment).

### Birth weight expressed as percentiles derived from birth weight standards

The INTERGROWTH-21st standards for birth weight are available only for gestational ages ≥24 + 0 weeks. Therefore, it was possible to derive percentiles from these standards only for 80% (12/15), 81% (26/32), and 100% (22/22) of newborns from pregnancies with intra-amniotic infection, with sterile intra-amniotic inflammation, and without intra-amniotic inflammation, respectively.

**Figure 2:**
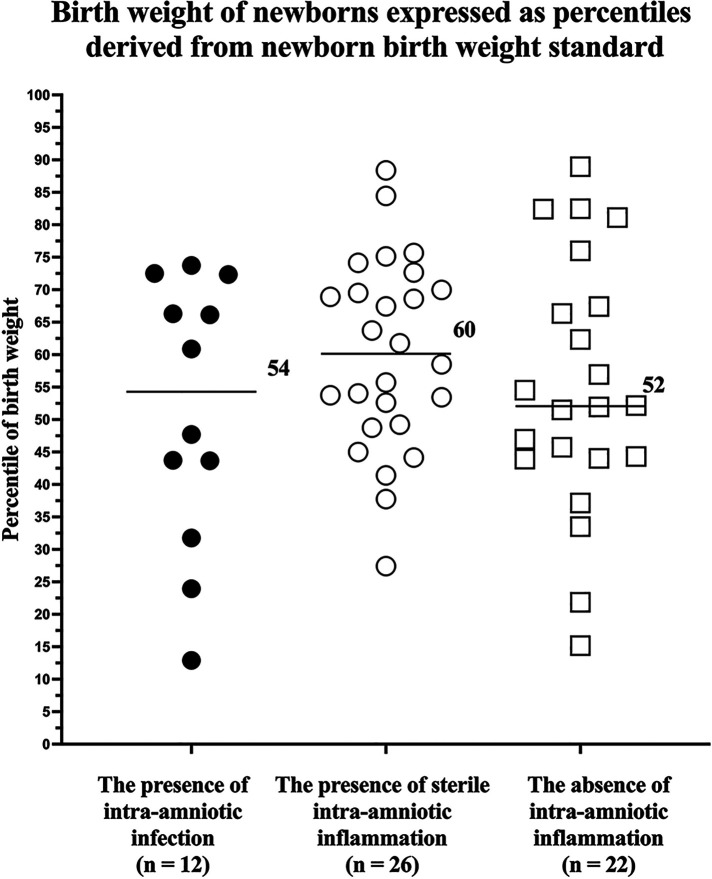
Comparison of newborn birth weight, expressed as percentiles derived from neonatal birth weight standards, from pregnancies with preterm labor with intact membranes prior to a gestational age of 35 weeks with the presence of intra-amniotic infection, presence of sterile intra-amniotic inflammation, and absence of intra-amniotic inflammation. Medians are marked.

No differences among the subgroup of women with intra-amniotic infection, with sterile intra-amniotic inflammation, and without intra-amniotic inflammation were identified in percentiles of newborn birth weight (the presence of intra-amniotic infection: median 54, IQR 35–71; the presence of sterile intra-amniotic inflammation: median 60, IQR 49–71; and the absence of intra-amniotic inflammation: median 52, IQR 44–70; *p* = 0.98; Figure 2) and in the rates of birth weight that were less than the 1st, 3rd, 10th, and 25th percentiles (Table 3).

**Table 3:** The rates of newborns with birth weights, expressed as percentiles derived from birth weight standards, that were less than or equal to the 1st, 3rd, 10th, and 25th percentiles from pregnancies with preterm labor with intact membranes prior to a gestational age of 35 weeks with the presence of intra-amniotic infection, presence of sterile intra-amniotic inflammation, and absence of intra-amniotic inflammation.

	<1st percentile	<3rd percentile	<10th percentile	<25th percentile
The presence of intra-amniotic infection (*n* = 12)	0 (0%)	*–*	0 (0%)	*–*	0 (0%)	–	2 (17%)	*p* = 0.64
The presence of sterile intra-amniotic inflammation (*n* = 26)	0 (0%)		0 (0%)		0 (0%)		0 (0%)	
The absence of intra-amniotic inflammation (*n* = 22)	0 (0%)		0 (0%)		0 (0%)		2 (10%)	

Variables are presented as number (%) and were compared using Cochran-Armitage test for trend.

## Discussion

The principal findings of the study carried out on women with PTL who delivered within seven days of admission were as follows: (i) no difference in percentiles of birth weight, derived from estimated fetal weight standard, was found among the subgroups of women with intra-amniotic infection, with sterile intra-amniotic inflammation, and without intra-amniotic inflammation; (ii) differences in the rates of birth weight that were less than the 10th and 25th percentiles, derived from the standard for estimated fetal weight, were identified among the subgroups of women with intra-amniotic infection, with sterile intra-amniotic inflammation, and without intra-amniotic inflammation; and (iii) no differences among the subgroups were revealed when percentiles were derived from the standard for birth weight.

In this study, no difference in the newborn birth weight, expressed in percentiles derived from the INTERGROWTH-21st standard for estimated fetal weight, was found among the subgroups of PTL pregnancies with intra-amniotic infection, with sterile intra-amniotic inflammation, and without of intra-amniotic inflammation. Nevertheless, the subgroup with intra-amniotic infection had a median percentile of only 29, whereas the medians of the subgroups with sterile intra-amniotic inflammation and without intra-amniotic inflammation reached 54 and 53, respectively. Irrespective of the absence of significant statistical differences among the subgroups, the presence of strong intra-amniotic inflammation (the median IL-6 levels in the amniotic fluid of the subgroup with intra-amniotic infection was 50,000 pg/ml) might have affected fetal growth when compared to the presence of weak intra-amniotic inflammation (the median IL-6 levels in the amniotic fluid of the subgroup with sterile intra-amniotic inflammation was 8,268 pg/ml) or the absence of these conditions. We do not want to overemphasize this observation; however, this hypothesis is further supported by the trend in the rates of newborns with a birth weight less than the 10th and 25th percentiles among the subgroups, with this trend being the highest in those with intra-amniotic infection, lower in those with sterile intra-amniotic inflammation, and the lowest in the absence of inflammation. The difference among the subgroups was revealed only in the cutoff values of the 10th percentile, which is commonly used by clinicians to identify fetuses and newborns with a diagnosis of small for gestational age (30), and the 25th percentile, which represents a cutoff value for the lowest quartile of birth weight that might identify and include a subset of newborns with a mild alteration of fetal growth. However, no association was found between the most severe alteration of fetal growth, represented by the cutoff values of the 1st and 3rd percentiles and both phenotypes of intra-amniotic inflammation. This is in line with observations from a recent animal study, where the intra-amniotic administration of endotoxin (*Escherichia coli*) at 20 days of gestation (the term is 22 days) led to a 20% and 22% decrease in birth and placental weights, respectively (15). Intra-amniotic administration of endotoxin in the rat model caused not only restriction of fetal and placental growth, but also decreased vascular density of the placenta. This observation is in line with findings from human studies that reported that maternal vascular malperfusion is related to alterations in fetal growth (31–36). In addition, a recent study by Jaiman et al. clearly showed that maternal vascular malperfusion is more frequent in PTL than in uncomplicated pregnancies with delivery at term (37).

Since intra-amniotic infection is considered a cause of cervical insufficiency with prolapsed fetal membranes or PTL rather than a consequence, it is likely that the fetal and placental exposure to this complication might be a long-lasting process compared to preterm prelabor rupture of membranes (PPROM), where this intra-amniotic complication is more likely a consequence. Collectively, the observations from this study are in line with those of our previous studies (20, 21). The results from our studies on the association between intra-amniotic inflammatory complications and cervical insufficiency with prolapsed fetal membranes (20), PPROM (21), and PTL suggest that the possible effect of intra-amniotic inflammation on fetal growth might be dependent on the following: (i) the length of exposure, (ii) the intensity of intra-amniotic inflammation, and iii) several unrecognized underlying endo- and exogenous factors (e.g., impaired placentation).

In this study, aside from the INTERGROWTH-21st standard for estimated fetal weight, the standard for newborn birth weight was also used to derive percentiles for birth weight. When this standard was applied, no differences in percentiles among the subgroups in the rates of birth weight less than the 1st, 3rd, 10th, and 25th percentiles were found. A similar phenomenon was observed in our previous studies (20, 21). We believe that the use of the standard for birth weight in studies such as ours is less appropriate because the standard for birth weight might be affected by the overrepresentation of pathological pregnancies resulting in iatrogenic or spontaneous preterm deliveries where impaired placentation might be expected (38). This limitation of birth weight standard might be seen mainly in the subset of newborns delivered before 33 weeks of gestation (27) and may not identify cases with mildly impaired fetal growth, particularly in newborns delivered very or extremely preterm. Since the subset of newborns delivered before 33 weeks of gestation represented 73% (50/69) of all newborns in this study, it is not surprising that no differences among the subgroups were observed when percentiles derived from the birth weight standard were used.

The main strength of this study was the thorough assessment of microbial invasion of the amniotic cavity based on a combination of three methods to detect microorganisms and/or their nucleic acids in amniotic fluid: (i) aerobic and anaerobic cultivation; (ii) specific PCR for *Ureaplasma* spp., *Mycoplasma hominis*, and *Chlamydia trachomatis*; and (iii) PCR for the 16S rRNA gene followed by sequencing. The employment of such an extensive approach offered us the opportunity to divide the subset of women with intra-amniotic inflammation into those with intra-amniotic infection and those with sterile intra-amniotic inflammation. Second, there is strong evidence that the latency interval between admission and delivery in pregnancy with PTL depends on the presence or absence of intra-amniotic inflammation (7, 8, 12). The latter is associated with a longer latency, which may take weeks instead of days, as is common in those with intra-amniotic inflammation (7, 8, 12). However, a long latency interval might represent a susceptible period for the alteration of fetal growth, independent of the initial intra-amniotic status, due to the possible effect of the antenatal course of corticosteroids (39, 40) or other endo- or exogenous stimuli. To eliminate the effect of a long latency interval, only PTL pregnancies with a meaningful temporal relationship between admission and delivery (≤7 days) were included in this study.

However, this study had some limitations. First, the small cohort of women with PTL used in this study was limited. The monocentric design of the study prevented us from using a larger sample size. Second, the population of women used in this study was homogeneous, involving only Caucasian participants. This potential shortcoming prevents the results of the study from generalizing to populations with broader ethnic and racial disparities. Finally, data regarding ultrasonographically estimated fetal weight obtained at the time of admission were not available. Therefore, we could not compare the percentiles of ultrasonographically estimated fetal weight at the time of admission, derived from the standard for estimated fetal weight, among the subgroups.

In conclusion, in this retrospective cohort study conducted on pregnancies with preterm labor and intact membranes prior to the gestational age of 35 weeks, the presence of intra-amniotic inflammatory complications was associated with a higher rate of newborns with birth weight less than the 10th and 25th percentile, when percentiles of birth weight were derived from the standard for estimated fetal weight.

## Data Availability

The raw data supporting the conclusions of this article will be made available by the authors, without undue reservation.
